# Plakoglobin and High-Mobility Group Box 1 Mediate Intestinal Epithelial Cell Apoptosis Induced by Clostridioides difficile TcdB

**DOI:** 10.1128/mbio.01849-22

**Published:** 2022-08-31

**Authors:** Yingxue Li, Wei Xu, Yutian Ren, Hung-Chi Cheung, Panpan Huang, Guneet Kaur, Chih-Jung Kuo, Sean P. McDonough, Susan L. Fubini, Stephen M. Lipkin, Xin Deng, Yung-Fu Chang, Linfeng Huang

**Affiliations:** a Division of Natural and Applied Sciences, Duke Kunshan University, Kunshan, Jiangsu, China; b Department of Biomedical Sciences, Jockey Club College of Veterinary Medicine and Life Sciences, City University of Hong Kong, Hong Kong SAR, China; c Department of Veterinary Medicine, National Chung Hsing University, Taiwan; d Department of Biomedical Sciences, College of Veterinary Medicine, Cornell University, Ithaca, New York, USA; e Department of Clinical Sciences, College of Veterinary Medicine, Cornell University, Ithaca, New York, USA; f Weill Cornell Medical College, New York, New York, USA; g Biotechnology and Health Centre, City University of Hong Kong Shenzhen Research Institute, Shenzhen, China; h Department of Population Medicine and Diagnostic Sciences, College of Veterinary Medicine, Cornell University, Ithaca, New York, USA; i Global Health Research Center, Duke Kunshan University, Kunshan, Jiangsu, China; The Hebrew University of Jerusalem

**Keywords:** *C. difficile*, TcdB, apoptosis, JUP, HMGB1, RNAi screen

## Abstract

Clostridioides difficile infection (CDI) is the leading cause of antibiotic-associated intestinal disease, resulting in severe diarrhea and fatal pseudomembranous colitis. TcdB, one of the essential virulence factors secreted by this bacterium, induces host cell apoptosis through a poorly understood mechanism. Here, we performed an RNA interference (RNAi) screen customized to Caco-2 cells, a cell line model of the intestinal epithelium, to discover host factors involved in TcdB-induced apoptosis. We identified plakoglobin, also known as junction plakoglobin (JUP) or γ-catenin, a member of the catenin family, as a novel host factor and a previously known cell death-related chromatin factor, high-mobility group box 1 (HMGB1). Disruption of those host factors by RNAi and CRISPR resulted in resistance of cells to TcdB-mediated and mitochondrion-dependent apoptosis. JUP was redistributed from adherens junctions to the mitochondria and colocalized with the antiapoptotic factor Bcl-X_L_. JUP proteins could permeabilize the mitochondrial membrane, resulting in the release of cytochrome *c*. Our results reveal a novel role of JUP in targeting the mitochondria to promote the mitochondrial apoptotic pathway. Treatment with glycyrrhizin, an HMGB1 inhibitor, resulted in significantly increased resistance to TcdB-induced epithelial damage in cultured cells and a mouse ligated colon loop model. These findings demonstrate the critical roles of JUP and HMGB1 in TcdB-induced epithelial cell apoptosis.

## INTRODUCTION

Clostridioides difficile infection (CDI) is the leading cause of antibiotic-associated intestinal disease. Antibiotic-mediated suppression of normal gut microbiota is strongly associated with colonization and proliferation of C. difficile (1). The clinical outcomes of this disease can range from asymptomatic carrier to diarrhea and potentially fatal pseudomembranous colitis. Due to the emergence of C. difficile hypervirulent strains ribotype 027 and 078, the morbidity and mortality rates of CDI have been rising globally over the past decades, posing a significant threat to public health (2–7). According to recent statistics from the CDC in the United States, C. difficile caused approximately 230,000 cases in 2017, of which 12,800 died, resulting in approximately $100 million in treatment costs (8). As a global health care problem, there is an urgent need to understand the infection mechanism and develop efficient CDI therapeutics.

C. difficile infection is mainly mediated by two exotoxins, C. difficile toxins A and B (TcdA and TcdB) (9–12). Both toxins are cytotoxic to cells, but TcdB causes more cell death and is responsible for severe illness (13). Therefore, we focused on TcdB in this work. Many studies have revealed that TcdB uses a multistep strategy to intoxicate host cells (14). It initially enters cells by binding to the receptors on the host intestinal surface and then is internalized via receptor-mediated endocytosis (15). Applications of genetic screens have identified several receptors for TcdB, including chondroitin sulfate proteoglycan 4 (CSPG4), poliovirus receptor-like protein 3 (PVRL3), and Frizzled family (FZDs), in various cell lines (16–18). However, those receptors are not universally expressed in all cell types. Thus, it is believed that TcdB could use multiple receptors at the same time or have specific receptors for different cell types. Recent studies also found that TcdB variants derived from different strains show varied binding affinities to the above receptors (19–22). Surprisingly, TcdB from the hypervirulent 027 strain has a low affinity to FZDs and PVRL3, which are expressed in epithelial cells, leading to the hypothesis that this TcdB variant may use other unknown receptors for cell entry.

Following the endocytosis of TcdB, acidification of the endosomes results in a conformational change of the toxin, leading to the insertion of the hydrophobic segments of TcdB into the endosome membrane and subsequent pore formation and translocation of the N-terminal glucosyltransferase domain (GTD) into the cytosol (23). By proteolytic cleavage of inositol hexakisphosphate (Insp6), GTD is released into the cytosol and inactivates Rho GTPase family proteins by glucosylation (24–26). The inactivation of Rho GTPases, key regulators of many essential cellular processes, causes cytopathic effects, characterized by cell rounding resulting from the disruption of the actin cytoskeleton, and cytotoxic effects, including programmed cell death and activation of inflammasomes (27). The cytotoxic attack could destroy the intestinal epithelium barrier and cause fluid accumulation in the intestinal lumen, tissue damage, and severe inflammation (28).

Cell death induced by TcdB appears to involve a complex scenario. Chumbler et al. discovered that TcdB causes apoptosis at low concentrations, and induces necrosis by reactive oxygen species (ROS) production at high concentrations (29, 30). Other studies showed TcdB is capable of inducing pyroptosis, a form of inflammatory cell death triggered by pyrin inflammasome activation and interleukin-1β (IL-1β) and IL-18 secretion (31–34).

Dissecting the cell death mechanism is critical for understanding CDI pathogenesis. Recent studies found that TcdB-induced apoptosis in intestinal epithelial cells (IEC) is more physiologically relevant *in vivo*. Saavedra et al. demonstrated that IEC apoptosis is critical to CDI but pyrin inflammasome-mediated pyroptosis is dispensable in mice (35). The same study also found that IEC apoptosis may restrict C. difficile spreading in early infection. Mileto et al. found that TcdB induces apoptosis in both colonic epithelial cells and stem cells located in the colonic crypts during CDI in mice (21). Furthermore, TcdB-induced apoptosis is mediated through caspase 3/7 and the mitochondrion-dependent intrinsic apoptotic pathway (36). However, the signaling pathway of TcdB-induced apoptosis is still unclear.

To study host factors involved in TcdB-induced apoptosis of epithelial cells, we carried out an RNA interference (RNAi) screen customized to a human colonic carcinoma epithelial cell line (Caco-2 cells) which could model IEC and is highly sensitive to apoptosis induced by TcdB. We identified several novel host factors that participated in TcdB-induced cell apoptosis. In particular, we report, for the first time, the essential role of junction plakoglobin (JUP), the γ-catenin, in epithelial cell apoptosis and the potential of inhibiting high-mobility group box 1 (HMGB1) for CDI therapy.

## RESULTS

### JUP and HMGB1 identified from an RNAi screen are involved in TcdB-induced apoptosis.

To identify cellular factors involved in TcdB-induced epithelial cell death, we conducted an RNAi screen on Caco-2 cells, a human colonic carcinoma cell line physiologically relevant to colon epithelium. Recombinant TcdB, produced according to a previous publication (37), was used for the RNAi screen (see Fig. S1A in the supplemental material). Previous studies showed that TcdB intoxication activates caspase-3 in epithelial cells and leads to cell death. We found that Caco-2 was the most sensitive cell line (compared to HeLa and 293T) to TcdB-induced caspase-3 activation measured by the Caspase-Glo 3/7 assay (Fig. S1B). Treatment with 0.01 nM TcdB can activate caspase-3 to more than 2-fold and cause a significant reduction in cell viability measured by the CellTiter-Glo assay. Treatment with TcdB or staurosporine (STS) can induce the cleavage of PARP-1, a target of activated caspase, confirming that TcdB has indeed activated caspase-3 (Fig. S1C). We conclude that Caco-2 cells could faithfully recapitulate TcdB-induced caspase activation with a very low concentration of TcdB (0.01 nM). Caco-2 serves as a model for intestinal epithelial cell apoptosis.

10.1128/mbio.01849-22.4 FIG S1 The establishment of the RNAi screen assay for TcdB. (A) SDS-PAGE and Coomassie blue staining showing purified TcdB. M, protein marker. (B) Three different cell lines (Caco-2, HeLa, and 293T cells) were treated with a serial concentration of TcdB for 18 h; cell viability and caspase 3/7 activity were assayed. Results are representative of three independent experiments performed in triplicate. Error bars represent the SEM. (C) Western blot analysis of PARP-1 cleavage for Caco-2 cells treated with a serial concentration of TcdB. Representative images are shown from one of three independent experiments. GAPDH was used as the loading control. Staurosporine (STS) was used as an apoptosis inducer. (D) A quantitative RT-PCR experiment was used to evaluate the knockdown efficiency of the indicated host genes in Caco-2 cells. Cells were transfected with 50 nM siRNA using Lipofectamine RNAiMAX, and total RNAs were harvested at 48 h posttransfection. Results are representative of three independent experiments performed in triplicate. Data are presented as the mean ± SEM. (E) Caco-2 cells were transfected with siRNAs targeting UGP2 and p22phox or siNC and then treated with the indicated amount of TcdB. Caspase 3/7 activation was measured at 18 h postintoxication. Results are representative of three independent experiments performed in triplicate. Error bars represent the SEM. Student’s *t* test; *, *P < *0.05; **, *P < *0.01; ***, *P < *0.001. Download FIG S1, TIF file, 0.5 MB. Copyright © 2022 Li et al. 2022 Li et al. https://creativecommons.org/licenses/by/4.0/ This content is distributed under the terms of the Creative Commons Attribution 4.0 International license.

We previously developed a technology for producing highly efficient and specific small interfering RNAs (siRNAs) from bacterial cells, named as pro-siRNAs (38, 39). A pro-siRNA-based siRNA library was cloned and produced using mRNAs extracted from Caco-2 cells. Caspase-Glo 3/7 assay was used as the screen indicator and 0.01 nM TcdB as the treatment. For the positive control, we chose two known host factors, UGP2 (UDP-glucose pyrophosphorylase) (18) and p22phox (a component of the NOX complex) (30), which have been demonstrated to be involved in TcdA- and TcdB-induced cell rounding or cell death. UGP2 is used by TcdA and TcdB to glucosylate GTPase. The knockout of UGP2 has been confirmed to render cells resistant to TcdA/TcdB-induced cell rounding (18). p22phox, a component of the NOX complex, silencing protects Caco-2 cells against TcdB-induced necrotic cell death (30). The siRNAs targeting those two genes achieved more than 90% knockdown of mRNA levels in Caco-2 cells (Fig. S1D). In TcdB-treated cells, caspase 3/7 activity was significantly reduced in p22phox and UGP2 siRNAs transfected cells compared with negative-control siRNA (siNC) transfected cells (Fig. S1E). This result also indicated that UGP2, as a key factor for the inactivation of Rho GTPase by TcdB, is involved in both cell rounding and apoptosis, and p22phox may have a novel role in the process of TcdB-induced apoptosis in addition to its role in TcdB-induced necrosis.

**FIG 1:**
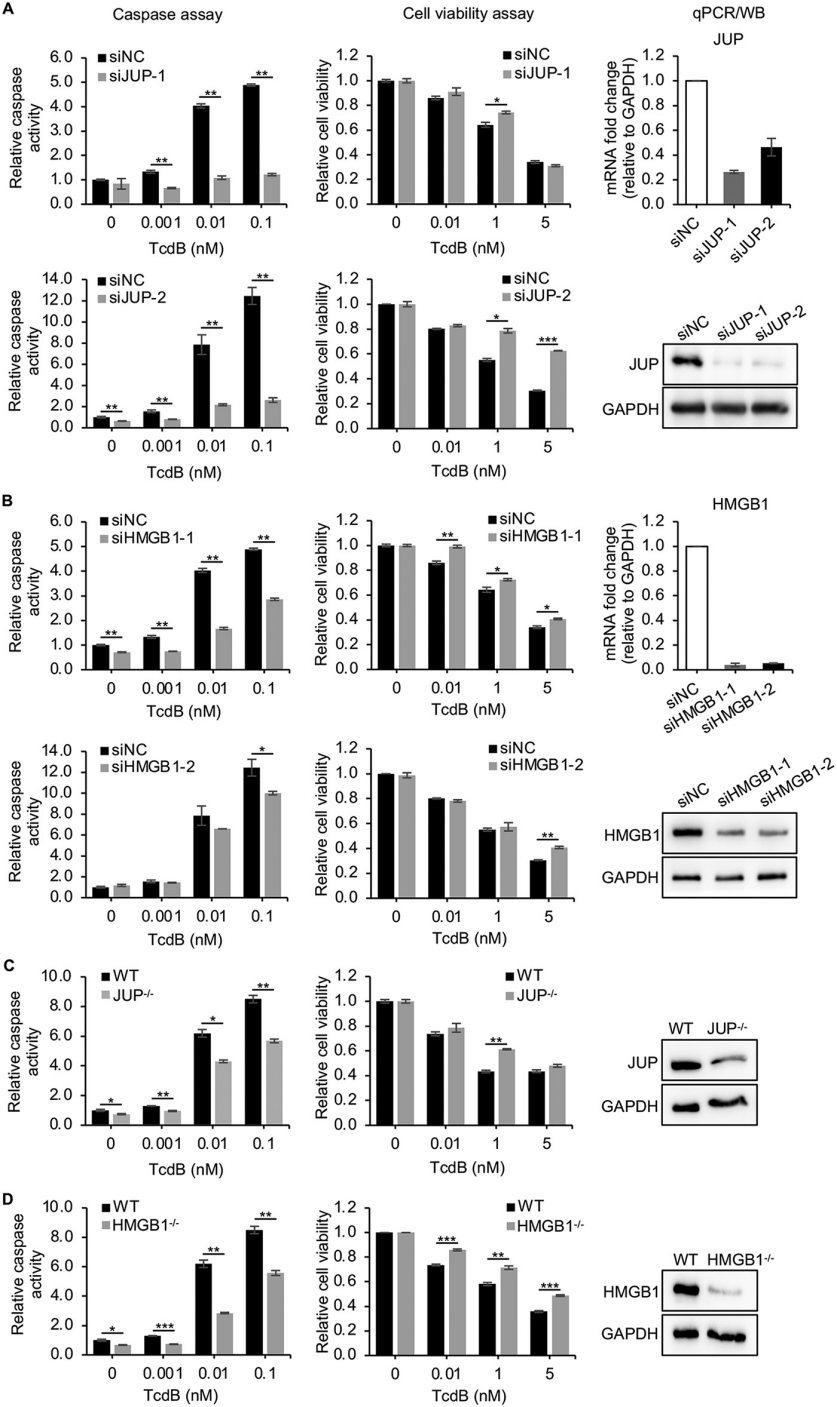
Knockdown of JUP and HMGB1 rescues TcdB-induced cell death. (A and B) Caco-2 cells were transfected with siNC or siRNAs targeting JUP (A) or HMGB1 (B) for 48 h and then treated with a serial concentration of TcdB for 18 h. Caspase 3/7 activation and cell viability were assayed. JUP and HMGB1 mRNA or protein levels were determined by quantitative RT-PCR (qPCR) or Western blotting (WB), respectively. GAPDH served as a loading control for WB. (C and D) The JUP^−/−^ (C) and HMGB1^−/−^ (D) knockout Caco-2 cells generated by CRISPR technology were treated with a serial concentration of TcdB for 18 h. Caspase 3/7 activation and cell viability were assayed. JUP and HMGB1 protein levels were determined by WB. All results are representative of at least three independent experiments performed in triplicate. Error bars represent the standard error of the mean (SEM). Student’s *t* test; *, *P* < 0.05; **, *P* < 0.01; ***, *P* < 0.001.

The schematic illustration of the pro-siRNA-based RNAi screen and validation process is shown in Fig. S2A. We performed a primary RNAi screen using the caspase activation assay with 1,920 pro-siRNA compounds. In the second round of pro-siRNA screening, an additional cell viability assay was used to confirm the caspase activation results. Two chemically synthesized siRNAs for each candidate were used to validate the pro-siRNA screen results. In the end, we identified 7 candidates (JUP, HMGB1, AHNAK, ITGB1, OGFR, SLK, SSRP1) that significantly reduced TcdB-induced caspase activation and cell death (Table S1). All the pro-siRNAs from the RNAi library induced silencing of these 7 candidates to less than 50% (Fig. S2B). For the synthetic siRNAs, quantitative reverse-transcription PCR (qRT-PCR) and/or Western blot experiments confirmed their knockdown efficiency, and the synthetic siRNAs also rescued TcdB-induced caspase activation and cell death (Fig. 1A and B and Fig. S3 and S4).

10.1128/mbio.01849-22.1 TABLE S1 Genes identified as involved in TcdB-mediated apoptosis. Download Table S1, DOCX file, 0.01 MB. Copyright © 2022 Li et al. 2022 Li et al. https://creativecommons.org/licenses/by/4.0/ This content is distributed under the terms of the Creative Commons Attribution 4.0 International license.

10.1128/mbio.01849-22.5 FIG S2 The pro-siRNA screen identified host factors for TcdB-induced apoptosis. (A) Schematic illustration of the RNAi screen for TcdB host factors and validation process. (B) The knockdown efficiency of pro-siRNAs of candidates tested by qRT-PCR. (C) The Z-score of the primary screening and the location of final selected candidates indicated by colored dots. (D) The ranking of 91 candidates based on the caspase 3/7 assay from the secondary screening. Download FIG S2, TIF file, 0.5 MB. Copyright © 2022 Li et al. 2022 Li et al. https://creativecommons.org/licenses/by/4.0/ This content is distributed under the terms of the Creative Commons Attribution 4.0 International license.

10.1128/mbio.01849-22.6 FIG S3 Validation of candidates using two sets of synthetic siRNAs. Caco-2 cells were transfected with two sets of synthetic siRNAs for each candidate and then exposed to TcdB for 18 h. Caspase 3/7 activation and cell viability were assayed. The knockdown efficiency of synthetic siRNAs was tested by qPCR. Results are representative of three independent experiments performed in triplicate. Error bars represent the SEM. Student’s *t* test; *, *P < *0.05; **, *P < *0.01; ***, *P < *0.001. Download FIG S3, TIF file, 0.6 MB. Copyright © 2022 Li et al. 2022 Li et al. https://creativecommons.org/licenses/by/4.0/ This content is distributed under the terms of the Creative Commons Attribution 4.0 International license.

10.1128/mbio.01849-22.7 FIG S4 Validation of candidates using two sets of synthetic siRNAs. Caco-2 cells were transfected with two sets of synthetic siRNAs for each candidate and then exposed to TcdB for 18 h. Caspase 3/7 activation and cell viability were assayed. The knockdown efficiency of synthetic siRNAs was tested by qPCR. Results are representative of three independent experiments performed in triplicate. Error bars represent the SEM. Student’s *t* test; *, *P < *0.05; **, *P < *0.01; ***, *P < *0.001. Download FIG S4, TIF file, 0.4 MB. Copyright © 2022 Li et al. 2022 Li et al. https://creativecommons.org/licenses/by/4.0/ This content is distributed under the terms of the Creative Commons Attribution 4.0 International license.

JUP and HMGB1 were among the top candidates according to the Z-score plot from the primary screen and the ranking of caspase 3/7 values from the secondary screen (Fig. S2C and D). Two synthetic siRNAs also confirmed that silencing of both genes largely inhibited TcdB-induced caspase 3/7 activation and significantly rescued TcdB-induced cell death (Fig. 1A and B). We constructed JUP and HMGB1 knockout cells to confirm the RNAi results using CRISPR technology. The expressions of JUP and HMGB1, detected by Western blotting, were reduced in CRISPR-treated cell lines (Fig. 1C and D). After TcdB treatment, the changes in caspase activity and cell viability of knockout cells, compared to that in wild-type cells (Fig. 1C and D), were consistent with the results obtained from siRNA experiments. Taken together, these data suggest the critical roles of JUP and HMGB1 in the process of TcdB-induced apoptosis.

### Both JUP and HMGB1 are involved in TcdB-induced and mitochondrion-dependent apoptosis.

To determine whether TcdB-induced caspase 3/7-dependent apoptosis is via the mitochondrion-mediated pathway in our experiments, we treated Caco-2 cells with 0.01 nM and 1 nM TcdB for 0 to 24 h. At various time points, adherent cells were pooled with floating cells and lysed. The cytosolic fractions, excluding mitochondria, were isolated according to a previously established method (40) and subjected to immunoblotting. As a result, we detected the release of cytochrome *c* from the mitochondria to the cytosol in a time- and toxin dose-dependent manner (Fig. S5A and B), which is consistent with the previous findings that TcdB mainly induces mitochondrion-mediated intrinsic apoptosis (36). We also detected the expression of JUP in the cytosolic fraction and found a potential cleavage product of JUP at late time points after TcdB exposure (Fig. S5B), which might be related to TcdB-induced apoptosis.

10.1128/mbio.01849-22.8 FIG S5 TcdB induced mitochondrion-dependent apoptosis. (A) Cytochrome *c* release was detected in Caco-2 cells after exposure to a serial concentration of TcdB for 24 h. Cytoplasmic fractions (excluding mitochondrial fractions) were isolated and then subjected to Western blot analysis. (B) Caco-2 cells were treated with 0.01 nM or 1 nM TcdB for 0 to 24 h. At each time point, cytoplasmic fractions were isolated and then subjected to Western blot analysis. Representative images are shown from one of two independent experiments. Download FIG S5, TIF file, 0.3 MB. Copyright © 2022 Li et al. 2022 Li et al. https://creativecommons.org/licenses/by/4.0/ This content is distributed under the terms of the Creative Commons Attribution 4.0 International license.

**FIG 2:**
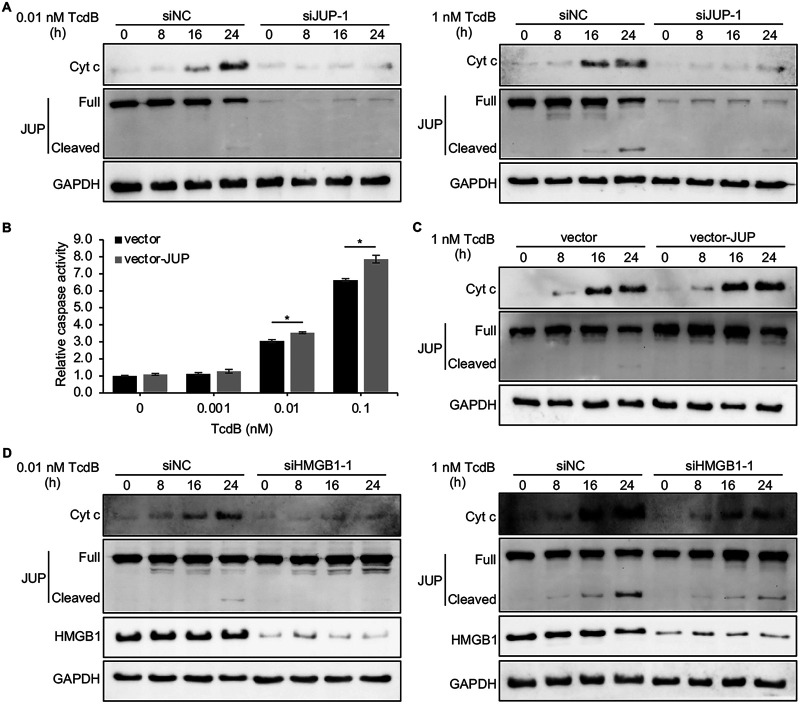
JUP and HMGB1 are involved in TcdB-induced and mitochondrion-dependent apoptosis. (A) Caco-2 cells were transfected with siRNA targeting JUP (siJUP-1) or siNC for 48 h, and then cells were treated with 0.01 nM (left) or 1 nM TcdB (right) for 0 to 24 h. Cytoplasmic fractions (excluding mitochondrial fractions) were collected at the indicated time points and subjected to Western blot analysis. Representative images are shown from one of two independent experiments. (B) Caco-2 cells were electroporated with control vector or vector expressing JUP (vector-JUP) and then treated with a serial concentration of TcdB for 18 h. Caspase 3/7 activation was then assayed. Results are representative of three independent experiments performed in triplicate. Error bars represent the SEM. Student’s *t* test; *, *P* < 0.05. (C) Caco-2 cells transfected with control vector or vector expressing JUP were treated with 1 nM TcdB for 0 to 24 h. At each time point, cytoplasmic fractions were obtained and then subjected to Western blot analysis. Representative images are shown from one of two independent experiments. (D) Caco-2 cells were transfected with siRNA targeting HMGB1 (siHMGB1-1) or siNC for 48 h, and then cells were treated with 0.01 nM (left) or 1 nM TcdB (right) for 0 to 24 h. At each time point, cytoplasmic fractions were obtained and then subjected to Western blot analysis. Representative images are shown from one of two independent experiments. GAPDH was used as the loading control. Cyt c, cytochrome *c*.

To confirm that JUP was involved in TcdB-induced and mitochondrion-mediated apoptosis, Caco-2 cells were transfected with either siRNA targeting JUP (siJUP-1) or siNC and then exposed to 0.01 nM or 1 nM TcdB for 0 to 24 h. The expression of JUP in cells transfected with siJUP-1 was almost completely inhibited (Fig. 2A). Cytochrome *c* release was barely detectable in the cells transfected with siRNA targeting JUP over the time course examined with 0.01 nM and 1 nM TcdB (Fig. 2A). These data suggest that inhibition of JUP blocks cytochrome *c* release caused by TcdB. We also used pcDNA3 vector to overexpress JUP in Caco-2 cells. Caco-2 cells transfected with empty or JUP overexpression vector (vector-JUP) were treated with a serial concentration of TcdB. JUP-overexpressed cells showed increased caspase activation compared with control cells when treated with TcdB at 0.01 and 0.1 nM (Fig. 2B). Immunoblotting confirmed the increased amount of cytochrome *c* release in Caco-2 cells transfected with vector-JUP (Fig. 2C).

To define whether HMGB1 is also involved in TcdB-induced and mitochondrion-mediated apoptosis, we performed the same cytochrome *c* release assay in Caco-2 cells transfected with siRNA targeting HMGB1 (siHMGB1-1). The expression of HMGB1 in cells transfected with siHMGB1-1 was almost completely inhibited (Fig. 2D). The results showed that in HMGB1-depleted cells, cytochrome *c* release into the cytosol from the mitochondria was largely reduced over the time course examined under both 0.01 nM and 1 nM TcdB treatment (Fig. 2D). In the meantime, the cleavage of JUP was also reduced in HMGB1-depleted cells at the late time course after TcdB exposure, indicating the potential function of HMGB1 in the regulation of JUP cleavage during TcdB-induced apoptosis. Taken together, those data indicate that both JUP and HMGB1 are involved in TcdB-induced and mitochondrion-dependent apoptotic pathway.

### JUP silencing blocks the cell death phenotype upon the stimulation of TcdB.

**FIG 3:**
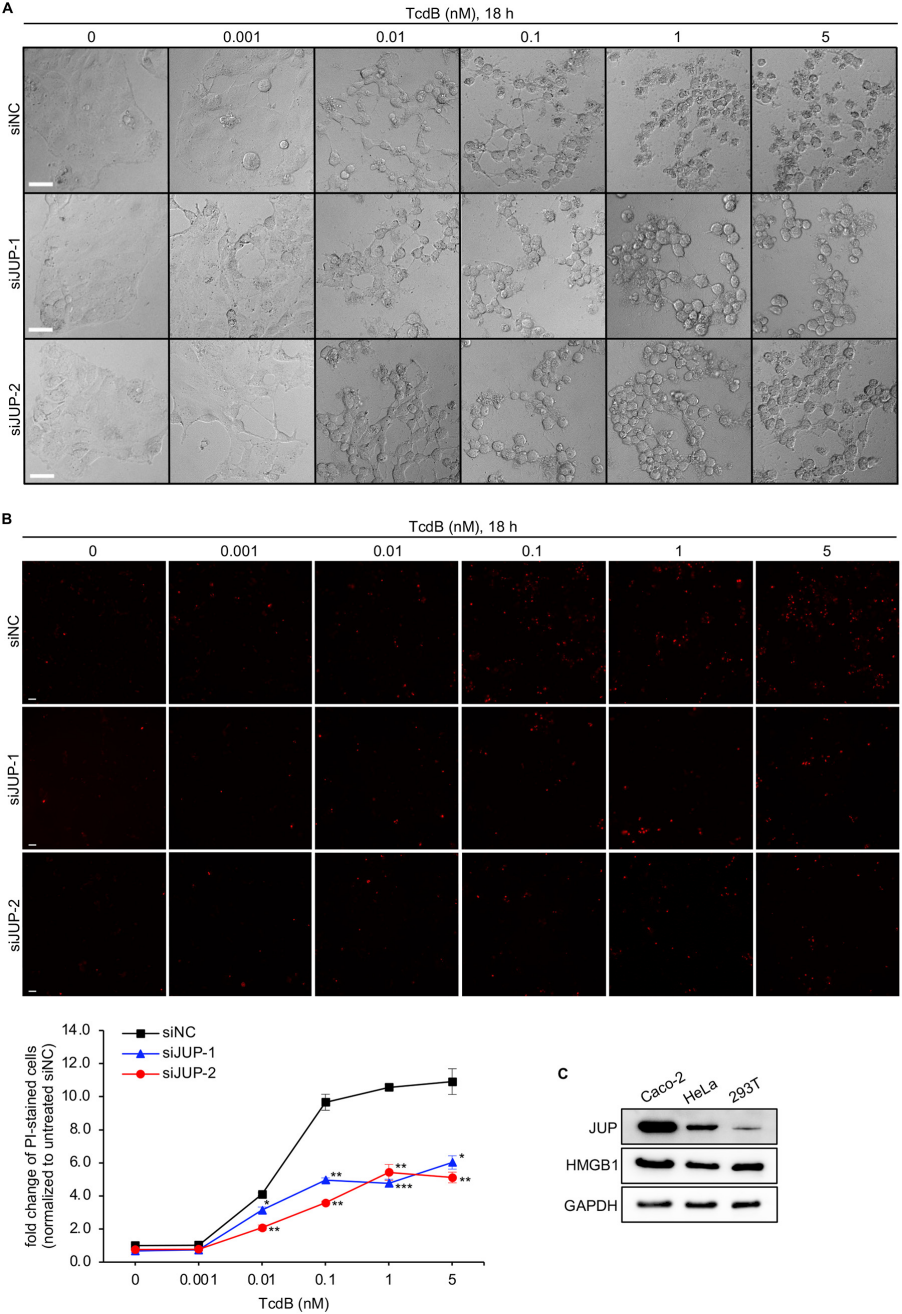
JUP silencing protects cells against TcdB-induced cell damage. (A) Caco-2 cells were transfected with two siRNAs targeting JUP (siJUP-1 and siJUP-2) or siNC for 48 h and then were treated with a serial concentration of TcdB for 18 h. Representative images from one of two independent experiments performed in triplicate show the cell morphology. Scale bar, 50 μm. (B) Cells were treated the same as in panel A but were stained with propidium iodide (PI) after 18 h of incubation with TcdB. Representative images from one of two independent experiments performed in triplicate show PI-stained dead cells. Scale bar, 50 μm. The percentage of PI-stained cells was analyzed with the HCS platform. Data are presented as the mean ± SEM. Asterisks indicate statistically significant differences between the experimental sample and the control group (siNC) at the same concentration of TcdB. Student’s *t* test; *, *P* < 0.05; **, *P* < 0.01; ***, *P* < 0.001. (C) The expression levels of JUP and HMGB1 were examined by immunoblot analysis of cell lysates from three different cell lines. GAPDH was used as the loading control.

JUP-silenced Caco-2 cells were less susceptible to TcdB-induced cell death, as shown by microscopic imaging analyses on cell morphology and cell death using propidium iodide (PI) staining (Fig. 3A and B). After treatment with TcdB at 18 h, cell debris was visible in siNC transfected cells starting at 0.1 nM TcdB, but not in siRNAs targeting JUP-transfected cells (Fig. 3A). However, the JUP siRNAs did not inhibit the cell rounding phenotype caused by TcdB treatment (Fig. 3A). The proportions of PI-positive cells (dead cells) were significantly reduced in cells transfected with JUP siRNAs compared with cells transfected with siNC (Fig. 3B).

Furthermore, we found that JUP is highly expressed in Caco-2 cells and much less expressed in HeLa and 293T cells (Fig. 3C). The sensitivity of three cell lines to TcdB-induced caspase 3/7 activation and cell death was positively correlated with the expression level of JUP since HeLa and 293T cells are not as sensitive as Caco-2 cells to TcdB-induced apoptosis (Fig. S1B). Those results suggest that JUP plays an essential role in the regulation of apoptosis in colon epithelial cells with a high expression level of JUP.

Taken together, these results suggest that JUP is required for cytochrome *c* release, which leads to apoptosis in TcdB-treated Caco-2 cells through an unknown mechanism. Meanwhile, JUP appears to be unrelated with TcdB-induced cytopathic effects (cell rounding).

### JUP is translocated into the mitochondria and colocalized with Bcl-X_L_ in cells treated with TcdB.

**FIG 4:**
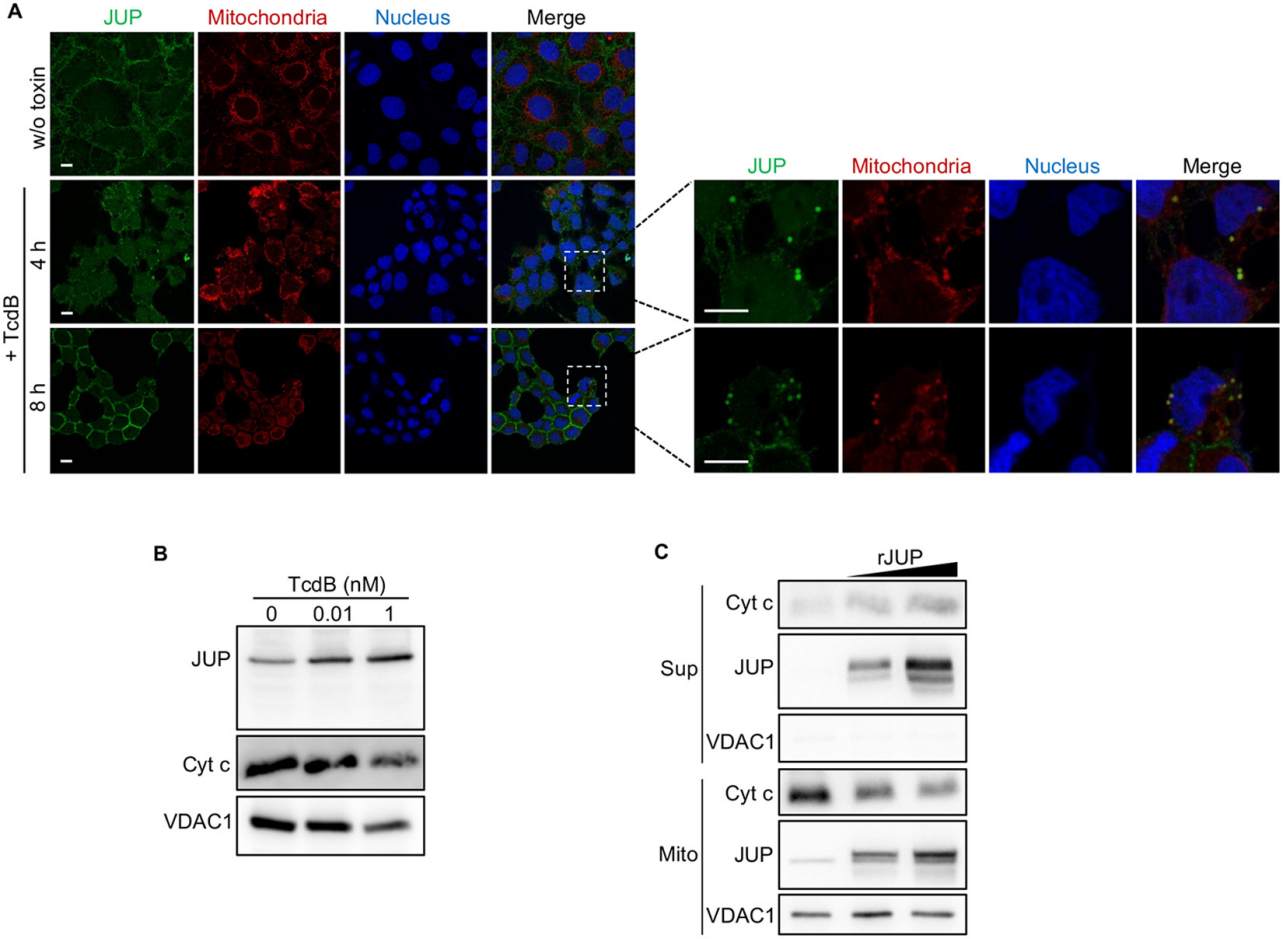
JUP is redistributed to mitochondria and permeabilizes mitochondria in cells after exposure to TcdB. (A) Caco-2 cells were incubated with 1 nM TcdB for 4 h and 8 h or without TcdB treatment (w/o toxin). At each time point, cells were stained with MitoTracker deep red at 37°C for 30 min and then stained with anti-JUP antibody. Representative images are from one of three independent experiments. All scale bars, 10 μm. (B) Caco-2 cells were either untreated or treated with 0.01 and 1 nM TcdB for 18 h. Mitochondria were isolated and subjected to immunoblot analysis. Representative images were from one of three independent experiments. (C) Immunoblots of cytochrome *c* (Cyt c) released into the reaction supernatant (Sup) from purified mitochondria (Mito) incubated with or without recombinant JUP (rJUP). Representative images are from one of three independent experiments. Voltage-dependent anion channel 1 (VDAC1) was used as the loading control for mitochondria.

In the intrinsic apoptotic pathway, the release of cytochrome *c* is caused by mitochondrial outer membrane permeabilization (MOMP), which is tightly controlled by Bcl-2 family members (41). Since the factors directly regulating cytochrome *c* release are in the mitochondria, we hypothesized that JUP could associate with mitochondria and mediate apoptosis. To test this hypothesis, we used confocal microscopy to study the localization and distribution of JUP in Caco-2 cells with or without TcdB treatment. In untreated cells, anti-JUP antibody staining showed that JUP is primarily present at the cell periphery, presumably closely associated with cell membrane and playing a role in cell-cell contact (Fig. 4A) (42). Treatment with 1 nM TcdB led to the initiation of cell rounding at 4 h and complete cell rounding at 8 h. At both time points, JUP signals were increased across the entire cytoplasmic space and, in some cells, formed cytoplasmic puncta, which are also colocalized with MitoTracker Red, a mitochondrial marker. The colocalization between JUP and mitochondria in cytoplasmic puncta was also observed in 293T cells transfected with Flag-tagged JUP after exposure to 1 nM TcdB for 24 h (Fig. S6).

10.1128/mbio.01849-22.9 FIG S6 Immunostaining of JUP and mitochondria in 293T cells. 293T cells were transfected with Flag-tagged JUP and then either untreated (w/o toxin) or treated with 1 nM TcdB for 24 h. 293T cells were stained with MitoTracker deep red at 37°C for 30 min and then stained with anti-Flag antibody. Representative images are from one of three independent experiments. Arrows indicate regions of colocalization. Scale bar, 10 μm. Download FIG S6, TIF file, 1.0 MB. Copyright © 2022 Li et al. 2022 Li et al. https://creativecommons.org/licenses/by/4.0/ This content is distributed under the terms of the Creative Commons Attribution 4.0 International license.

To further confirm the translocation of JUP from the cell periphery to mitochondria, we isolated the mitochondria from cells treated with or without TcdB. The immunoblot result indicated that the JUP proteins in the mitochondria fraction are increased in a dose-dependent manner in the cells treated with 0.01 and 1 nM TcdB compared to untreated cells (Fig. 4B). However, we did not detect the cleavage fragment of JUP in the mitochondrion fraction from the cells treated with TcdB, suggesting the full-length JUP protein might target the mitochondria and increase the permeability of mitochondrial membrane, resulting in cytochrome *c* release and subsequent caspase activation. To provide direct evidence for that hypothesis, we incubated purified mitochondria with recombinant full-length JUP proteins. The JUP protein treatment induced the release of cytochrome *c* to the supernatant and the reduction of cytochrome *c* in the purified mitochondria (Fig. 4C). These data support that the translocation of JUP into the mitochondria coincides with TcdB-induced apoptosis and that JUP could directly induce mitochondrial membrane permeabilization and cytochrome *c* release.

**FIG 5:**
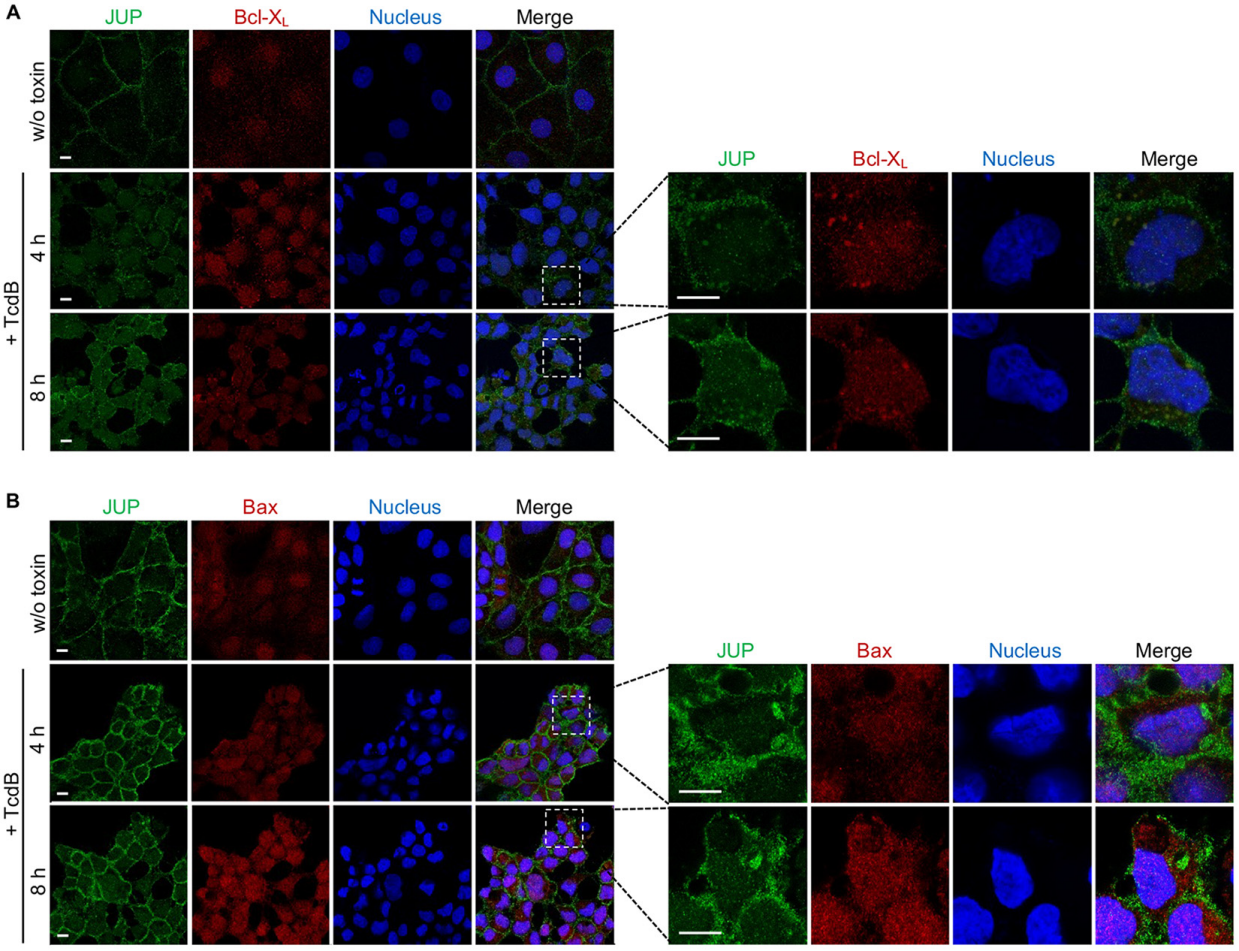
JUP is colocalized with Bcl-X_L_ in cells after exposure to TcdB. (A and B) Caco-2 cells were either untreated (w/o toxin) or treated with 1 nM TcdB for 4 h and 8 h. Cells were costained for JUP and Bcl-X_L_ (A) or Bax (B). Representative images are from one of three independent experiments. All scale bars, 10 μm.

The antiapoptotic members of the Bcl-2 family, Bcl-2 and Bcl-X_L_, inhibit MOMP by binding to and sequestering proapoptotic Bax/Bak proteins. Therefore, we tested if JUP could be linked to any Bcl-2 family members. Immunofluorescent costaining of JUP with Bcl-X_L_ or Bax showed colocalization of JUP and Bcl-X_L_ in the cytoplasmic puncta in TcdB-treated cells (Fig. 5A). No colocalization was observed between JUP and Bax (Fig. 5B). However, we could not detect a direct interaction between JUP and Bcl-X_L_ by coimmunoprecipitation (data not shown). These results suggest that JUP might be associated with the antiapoptotic protein Bcl-X_L_ through an indirect interaction. Such association might promote the activation of Bax/Bak, which may subsequently cause cytochrome *c* release.

### Glycyrrhizin, an inhibitor of HMGB1, alleviates TcdB-induced epithelial damage in cultured cells.

Previous studies have reported that TcdA/B could induce the release of HMGB1 from the nucleus into the cytosol, and eventually into the extracellular environment, where HMGB1 further stimulates acute inflammation in intestinal tissue (43–45). Consistently, after Caco-2 cells were exposed to TcdB, we also detected the presence of HMGB1 in the extracellular medium in a time- and dose-dependent manner, while the intracellular level of HMGB1 remained stable (Fig. S7). Our findings further support that HMGB1 plays a vital role in TcdB-induced apoptosis and could serve as a potential therapeutic target for treating CDI.

10.1128/mbio.01849-22.10 FIG S7 HMGB1 is released into culture medium after stimulation by TcdB. Caco-2 cells were treated with 0.01 nM or 1 nM TcdB for 0 to 24 h. At each time point, the whole-cell lysates and proteins from the medium were obtained and then subjected to Western blot analysis. Representative images are shown from one of two independent experiments. GAPDH was used as the loading control. Download FIG S7, TIF file, 0.2 MB. Copyright © 2022 Li et al. 2022 Li et al. https://creativecommons.org/licenses/by/4.0/ This content is distributed under the terms of the Creative Commons Attribution 4.0 International license.

**FIG 6:**
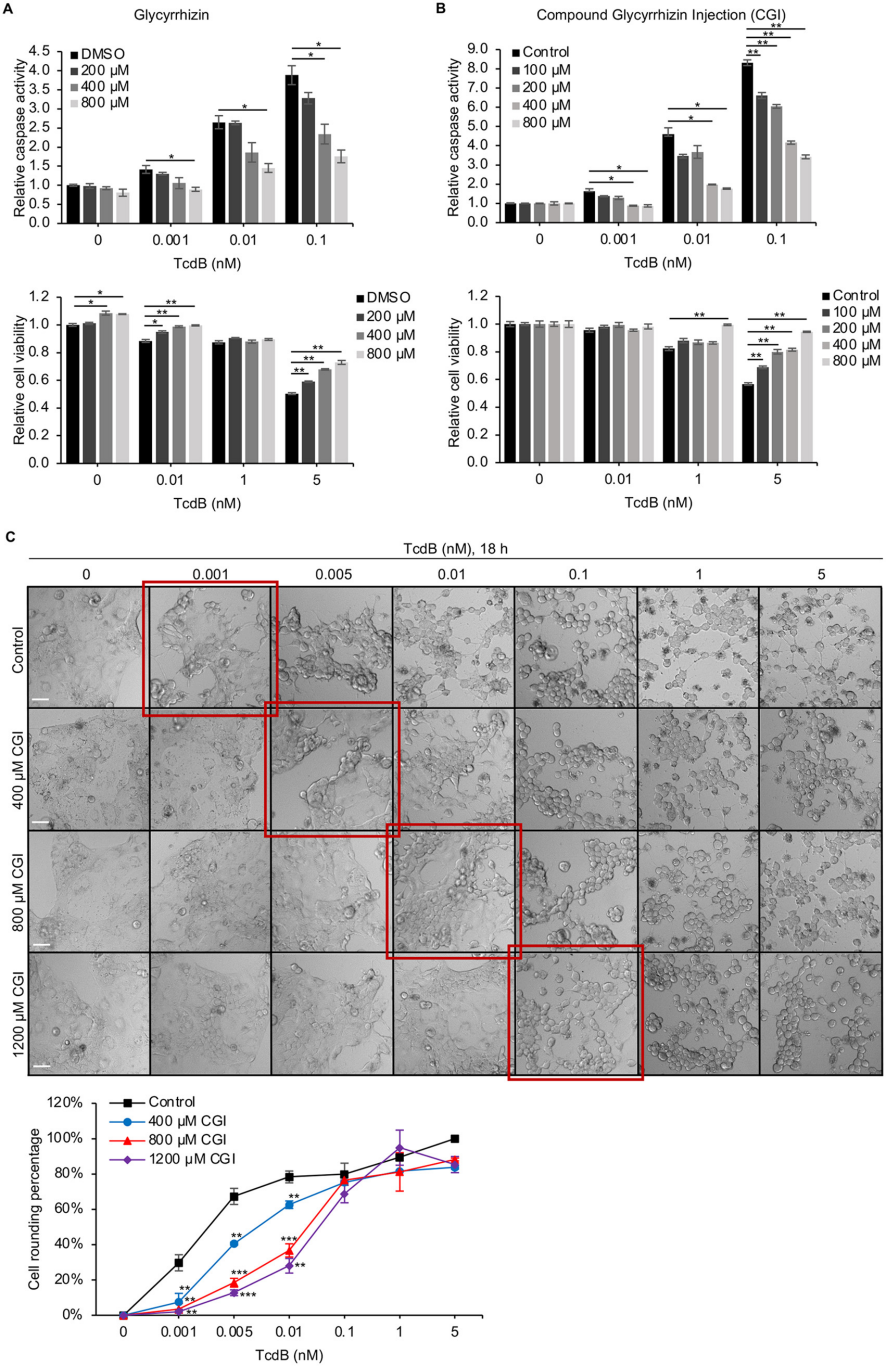
Glycyrrhizin pretreatment alleviates TcdB-induced epithelial damage in cultured cells. (A and B) Caco-2 cells were pretreated with glycyrrhizin (A) or compound glycyrrhizin injection (CGI) (B) and then treated with TcdB for 18 h; caspase activation and cell viability were assayed. Results are representative of three independent experiments performed in triplicate. (C) The protective effects of CGI on TcdB-induced cytopathic effect. Caco-2 cells were pretreated with CGI for 2 h before being treated with a serial concentration of TcdB for 18 h. Representative images from one of three independent experiments performed in triplicate show the cell rounding effect. Scale bar, 50 μm. The percentage of rounded cells was analyzed using the HCS platform. Error bars represent the SEM. Asterisks indicate statistically significant differences between the experimental sample and the control group at the same concentration of TcdB. Student’s *t* test; *, *P* < 0.05; **, *P* < 0.01; ***, *P* < 0.001.

Glycyrrhizin (also called glycyrrhizic acid), as a well-proven inhibitor of HMGB1, was used to determine if glycyrrhizin treatment could alleviate TcdB-induced apoptosis. Glycyrrhizin is a plant glycoside extracted from the roots of the licorice plant. It has two isomers:18β- and 18α-glycyrrhizin derived from 18β-glycyrrhizin by isomerization. Naturally, the root of the licorice has more content of 18β-glycyrrhizin. The purified glycyrrhizin we used in this study is extracted from the licorice with >98% purity (Chengdu Mansite Pharmaceutical Co. Ltd.). Compound glycyrrhizin injection (CGI; Minophagen Pharmaceutical Co. Ltd., Japan), an approved drug in China, is the second generation of glycyrrhizin as a compound preparation of 18β-glycyrrhizin. The injection is prepared with glycyrrhizin as the main active ingredient, with glycine and cysteine hydrochloride as additional ingredients to increase the drug stability, solubility, and safety. Both purified glycyrrhizin and CGI were used in our study to assess their protective effects against TcdB. Caco-2 cells were pretreated with a serial concentration of either purified glycyrrhizin or CGI and then exposed to different concentrations of TcdB ranging from 0.001 nM to 5 nM. Caspase and cell viability assays showed that both treatments significantly reduced TcdB-induced caspase activation and rescued TcdB-induced cell death in a dose-dependent manner compared with the cells pretreated with control buffers (Fig. 6A and B). In addition, Caco-2 cells pretreated with CGI showed a dose-dependent increase in resistance to TcdB-induced cell rounding (Fig. 6C), and the highest concentration of CGI pretreatment group had an approximately 20-fold resistance to TcdB compared with cells pretreated with control buffer according to high-content imaging analysis. These data showed that glycyrrhizin pretreatment significantly reduced TcdB-induced cell rounding and cell death.

### Glycyrrhizin alleviates TcdB-induced epithelial damage in a mouse colon ligation loop model.

**FIG 7:**
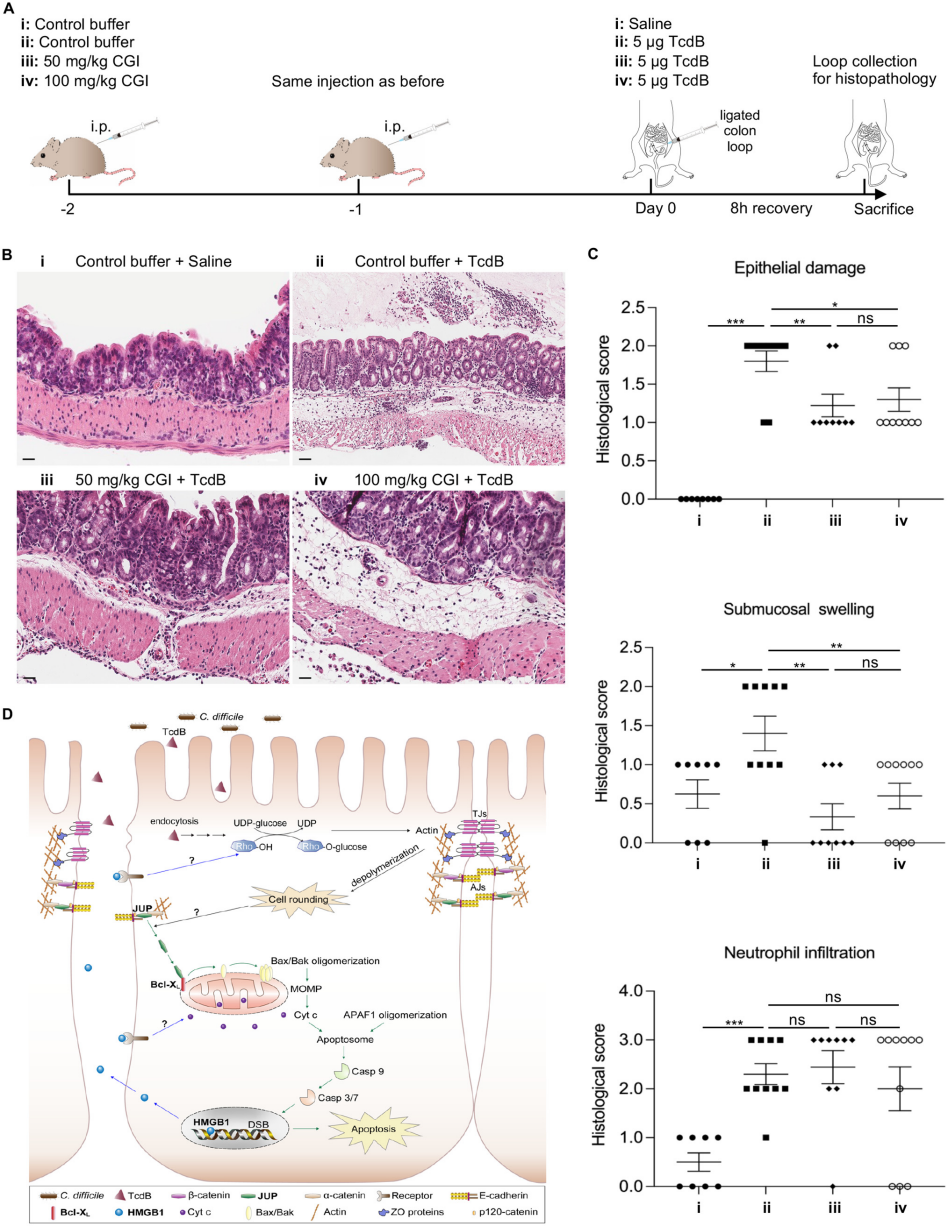
Glycyrrhizin pretreatment alleviates TcdB-induced epithelial damage in a mouse colon ligation loop model. (A) Schematic drawing illustrating that mice were injected daily with CGI or control buffer intraperitoneally (i.p.) for 2 days before TcdB or saline was injected into the ligated colonic segments; then the mice were euthanized, and the colon loops were collected for histopathology analysis. (B) Representative H&E-stained colon sections from each group. Scale bar, 50 μm. (C) The histological scores of epithelium damage, submucosal swelling, and neutrophil infiltration were assessed for different groups. Data are presented as the mean ± SEM. ANOVA; *, *P* < 0.05; **, *P* < 0.01; ***, *P* < 0.001. (D) A model for the potential functions of JUP and HMGB1 in TcdB-intoxicated intestinal epithelium. The green arrows indicate the potential signaling pathway triggered by dislocated JUP, which is possibly associated with the disruption of cell-cell contacts induced by TcdB. The blue arrows indicate the release of HMGB1 from the nucleus into the extracellular environment at the late apoptotic stage. It could bind to the specific receptor on the cell surface, transduce the signaling, and further regulate cell rounding and the mitochondrion-mediated apoptotic pathway. TJs, tight junctions; AJs, adherens junctions; Rho, Rho GTPase family proteins; MOMP, mitochondrial outer membrane permeabilization; DSB, double-strand break; Cyt c, cytochrome *c*; Casp, caspase; APAF1, apoptotic protease-activating factor 1.

To further test the effects of glycyrrhizin *in vivo*, we used a colon ligation loop model in mice to mimic C. difficile infection (46). The CGI has better solubility and safety than purified glycyrrhizin and, as an approved drug, can directly guide medication; therefore, we used CGI in the mouse model experiment. We used 50 mg/kg of body weight (one-tenth of the 50% lethal dose [LD_50_] provided in the drug instructions) as a starting dose and 100 mg/kg as a higher dose in the mouse experiment. As shown in Fig. 7A, mice were injected intraperitoneally with CGI at 50 and 100 mg/kg doses before the surgery. TcdB was directly injected into the lumen of ligated colon segments. After 8 h of treatment with TcdB, the ligated colon tissues were collected for histological analysis. The hematoxylin and eosin (H&E) staining results revealed that the ligated colon tissue treated by only TcdB presented pseudomembranous colitis in the lumen, severe submucosal swelling, pervasive neutrophil infiltration, and smooth muscle vacuolization, compatible with hydropic degeneration (Fig. 7B). The glycyrrhizin-injected mice had less severe lesions than the TcdB-only treatment group (Fig. 7B). The histological scoring for epithelial damage also revealed that TcdB caused severe damage to the epithelial layer, and glycyrrhizin injection significantly alleviated such damage (Fig. 7C). The histological scoring also revealed that the glycyrrhizin injection alleviated the submucosal swelling caused by TcdB, but not the neutrophil infiltration in colon tissue (Fig. 7C), suggesting that the protective effects of glycyrrhizin are limited to the epithelial integrity and submucosal swelling. The higher dose of glycyrrhizin pretreatment (100 mg/kg) did not produce a better protective effect against TcdB-induced epithelial damage compared to the lower dose of glycyrrhizin pretreatment (50 mg/kg) as shown in Fig. 7B.

Both the cell line and animal experiments suggest that glycyrrhizin pretreatment can protect colon epithelium against TcdB-induced damage. These findings support HMGB1 as a potential drug target using an approved drug of HMGB1 inhibitor, glycyrrhizin, for treating CDI.

## DISCUSSION

This study aimed to identify host factors involved in TcdB-induced apoptotic cell death using a novel RNAi screen approach. We selected Caco-2 cells as the model for colon epithelium, which is highly sensitive to TcdB-induced apoptosis. We created a bacterium-produced RNAi library, customized to Caco-2 cells, based on the pro-siRNA technology, and used caspase 3/7 and cell viability assays as the readouts for the RNAi screen. Our screen revealed multiple host factors that participated in TcdB-induced apoptosis.

We identified a novel host factor, JUP, required for TcdB-induced apoptosis and revealed a potential link between JUP and mitochondria. JUP, also known as γ-catenin, is a member of the armadillo protein family and has a very similar structure to the well-known β-catenin. JUP is a component of both adherens junctions and desmosomes, two structures that mediate cell-cell adhesion at the basolateral surfaces of polarized epithelia and help maintain structural integrity (47). Adherens junction complexes in epithelial cells are composed of E-cadherin, various catenins (e.g., α-, β- or γ-catenin), and the actin cytoskeleton. The extracellular domain of E-cadherin binds to other cadherins on adjacent cells to hold cells together, and the intracellular domain interacts with either β- or γ-catenin, which then connects to α-catenin and actin filament, forming a tight cell-cell connection. In desmosomes, the desmosomal cadherins consist of desmocollins and desmogleins, two transmembrane proteins that form dimers with the same desmosomal cadherins on neighboring cells. Their C-terminal cytoplasmic domains bind to plakophilin and JUP, both of which interact with desmoplakin, connecting to the cellular intermediate filament. The cadherin-catenin complex also plays essential roles in the regulation of cell proliferation, migration, survival, etc. (48). The essential role of JUP in tissue and body formation is evidenced by organ defects caused by the depletion of JUP, especially in the heart and skin (49).

The involvement of cell junctions, including tight junctions just beneath the apical surface of epithelial cells and subjacent adherens junctions, in the actions of TcdA/B has been noticed before. Previous studies showed that TcdA and TcdB could cause increased paracellular permeability associated with the displacement of occludin, ZO-1, or ZO-2 from the tight junctions, presumably resulting from the disorganization of F-actin by Rho inactivation (50, 51). Leslie et al. found that TcdA disrupts the paracellular barrier function in human intestinal organoids by inducing the dislocation of E-cadherin and tight junction proteins to the epithelial apical surface (52). Mileto et al. discovered that TcdB causes severe disruption of β-catenin/E-cadherin in colonic epithelia of mice infected with TcdB-producing C. difficile strains (21). A study found that the JUP mRNA level is upregulated in host fecal mRNA transcript expression profiling data from patients with CDI (53). However, no previous study identified a functional role of JUP in CDI.

In our study, we found that reduced JUP expression decreases the sensitivity of the Caco-2 cell line to TcdB-induced apoptosis, while its ectopic overexpression increases its sensitivity. Our finding is consistent with a previous study which demonstrated that JUP deficiency in keratinocytes protects cells from apoptosis induced by DNA-damaging apoptotic stimuli (54). We further explored the molecular mechanism of JUP in TcdB-induced apoptosis. JUP, like its homolog β-catenin, has also been reported to act in signal transduction by interacting with intracellular partners (55–57). Since the factors associated with cytochrome *c* release are mainly located in mitochondria, we examined the effects of TcdB on JUP distribution. Surprisingly, we found that JUP-containing intracellular bodies also have mitochondrial markers (Fig. 4A). The enrichment of JUP in the mitochondria after TcdB stimulation was also confirmed by immunoblotting on proteins isolated from a purified mitochondrial fraction (Fig. 4B). An *In vitro* experiment also verified the ability of JUP to stimulate the release of cytochrome *c* from mitochondria (Fig. 4C).

Furthermore, we observed colocalization between JUP and Bcl-X_L_, but not Bax (Fig. 5A and B). We speculate that JUP is required for an intracellular signaling event that leads to mitochondrion-dependent apoptosis in colon epithelial cells, and our model is shown in Fig. 7D. The cytopathic effect, cell rounding, caused by TcdB, appears to be an upstream event of apoptosis since the knockdown of JUP does not affect cell rounding (Fig. 3A). The disruption of cell-cell contacts could have triggered JUP translocation to the cytosol and recruitment to the mitochondria. JUP could serve as a proapoptotic factor similar to other non-Bcl-2 family proteins, which have been reported to induce MOMP, such as the members of the gasdermin protein family exhibiting pore-forming activity upon cleavage. It has been reported that during mitochondrial apoptosis, caspase 3-mediated cleavage of gasdermin E (GSDME) liberates a pore-forming N-terminal fragment that not only can promote plasma membrane permeabilization during apoptotic cell death, but also can localize to the mitochondria and permeabilize the mitochondria to potentiate apoptosis (58). Therefore, it is conceivable that JUP, after the break of adherens junctions, plays a proapoptotic role in the mitochondria. The full-length JUP proteins could function similarly to GSDME, as suggested by our experiment (Fig. 4C). Subsequently, JUP may affect Bcl-X_L_ and activate the proapoptotic factor Bax/Bak, resulting in the release of cytochrome *c* and the activation of apoptosome in the mitochondria. Collectively, our results suggest that in addition to its role in cell adhesion, JUP can also promote mitochondrial permeabilization and cytochrome *c* release during apoptosis. However, the exact mechanism of JUP activation leading to apoptosis remains to be uncovered.

We also screened out HMGB1 as a host factor for TcdB toxicity, consistent with a previous study (45). Genetic disruption of HMGB1 expression through siRNA knockdown or CRISPR/Cas9 mutagenesis confers resistance to TcdB-induced cell death, evidenced by reduced caspase 3/7 activation and cytochrome *c* release (Fig. 1B and D and Fig. 2D), supporting that HMGB1 is involved in the TcdB-induced and mitochondrion-dependent apoptosis pathway.

HMGB1 is a highly conserved DNA binding partner that is typically localized in the nucleus of almost all eukaryotic cells to act as a nuclear cofactor in transcription regulation (59). In addition to its intracellular functions, HMGB1 also can be released into the extracellular environment in two distinct ways: secreted actively by live immune cells such as macrophages or released passively by dead or dying cells. Extracellular HMGB1 can activate inflammatory responses and contribute to many inflammatory diseases by binding to cell-specific receptors (60, 61). Extracellular HMGB1 stimulated by TcdA mediates acute intestinal inflammation (44). Recent studies determined that HMGB1 could be released into the extracellular milieu after TcdB treatment and act as a necrosis marker (29, 30). A study has found that HMGB1 and its B-box domain are capable of increasing the permeability of intestinal epithelia and impairing the intestinal barrier by binding to RAGE (receptor for advanced glycation end products) receptor to initiate a signaling cascade that ultimately leads to inducible nitric oxide synthase (iNOS) production (62). Our results indicate that HMGB1 is vital for both cytotoxic and cytopathic effects of TcdB (Fig. 1B and D and Fig. 6A to C), and the release of HMGB1 into the extracellular space is confirmed in cell line experiments (Fig. S7). In summary, HMGB1 could be released into the extracellular environment from TcdB-induced apoptotic cells at a very late stage, and it could also regulate both cell rounding and cell death through intracellular signaling pathways, as shown in Fig. 7D. These findings suggest that targeting HMGB1, including its secreted portions, might be an effective strategy to reduce colon epithelial damage caused by both TcdA and TcdB.

Glycyrrhizin, a Chinese medicine compound from the root of the licorice plant, has been used to treat chronic hepatitis for decades in Japan and China (63). This compound has been reported to have various pharmacological effects, including anti-inflammatory, antiviral, antitumor, and hepatoprotective activities. It has been identified as an HMGB1 inhibitor that binds directly to both HMG boxes in HMGB1 (64). Glycyrrhizin has a protective effect on TcdA-induced acute intestinal inflammation and endoplasmic reticulum (ER) stress (43, 44). In this study, we determined that glycyrrhizin pretreatment can curtail TcdB-induced cell damage according to the following changes: the robust cell viability increase, caspase activation decrease, and cell rounding resistance (Fig. 6A to C). Promising results against TcdB-induced colon damage were obtained when using 50 mg/kg of the glycyrrhizin injection in a mouse colon ligation loop model (Fig. 7B and C). Glycyrrhizin could be inhibiting extracellular HMGB1. Our results suggest that the therapeutic effects of glycyrrhizin for CDI are worth further study, e.g., in an animal infection model with the postinfection treatment of the drug.

In summary, JUP and HMGB1 are host factors for TcdB-induced cell apoptosis, which involves cytochrome *c* release from the mitochondria and caspase activation. A model for the actions of JUP and HMGB1 is shown in Fig. 7D. For the first time, JUP is shown to play an essential role in regulating cell death of colon epithelial cells and in CDI. HMGB1 affects both cell rounding and cell death and is secreted, while JUP is involved only in an intrinsic cell death pathway. New treatment strategies for CDI could be developed based on targeting those host factors.

## MATERIALS AND METHODS

### Antibodies, inhibitors, and constructs.

Primary antibodies for the following genes were used: PARP-1 (Cell Signaling Technology [CST] USA, no. 9542), γ-catenin (CST, no. 2309), HMGB1 (Abcam UK, ab18256), Bcl-X_L_ (Santa Cruz Biotechnology [SCB] USA, sc-8392), Bax (SCB, sc-7480), cytochrome *c* (SCB, sc-13156), VDAC1 (SCB, sc-390996), and GAPDH (SCB, sc-47724). The horseradish peroxidase (HRP)-conjugated anti-mouse IgG (31430), anti-rabbit IgG (31460), Alexa-Fluor 488 donkey anti-rabbit IgG (A-21206), and Alexa-Fluor 594 goat anti-mouse IgG (A-11032) secondary antibodies were purchased from Thermo Fisher Scientific. Glycyrrhizin was purchased from Chengdu Mansite Pharmaceutical Co. Ltd. (Chengdu, Sichuan, China). Compound glycyrrhizin injection (CGI) was from Minophagen Pharmaceutical Co. Ltd. (Shinjuku, Tokyo, Japan). Flag-tagged full-length human γ-catenin construct in the pcDNA3 vector was obtained from Addgene (plasmid no. 16827).

### Cell lines.

The human colorectal cancer Caco-2 cells, HeLa cells and HEK293T cells were cultured in Dulbecco’s modified Eagle’s medium (DMEM, Gibco) supplemented with 10% fetal bovine serum (FBS, Gibco) at 37°C with 5% CO_2_ as previously described (10, 39).

### TcdB recombinant protein.

Recombinant TcdB (from C. difficile strain 630) was cloned into pHis1522 vector (MoBiTec), produced in Bacillus megaterium cells as previously described (37), and purified using nickel affinity chromatography and size exclusion chromatography.

### Cell viability and caspase assays.

Cell viability was measured with the CellTiter-Glo luminescent cell viability assay (Promega) using a microplate reader according to the manufacturer’s instructions. Apoptosis was quantified by measuring caspase 3/7 activation of a luminescent signal using Caspase-Glo 3/7 assay (Promega).

### siRNA reverse transfection.

All chemically synthesized siRNAs were ordered from GenePharma or RiboBio. The sequences of siRNAs are listed in Table S2. The negative control siRNA (siNC) is a standard non-targeting siRNA (GenePharma). An siRNA reverse transfection method was used in this study, as it is known to have a high knockdown efficiency for Caco-2 cells. For transfection into a 96-well plate, 0.5 μL chemically synthesized siRNA (10 μM stock, final 50 nM) was diluted in 25 μL Opti-MEM. Lipofectamine RNAiMAX (Thermo Fisher Scientific) was prepared in 25 μL Opti-MEM at 0.2 μL per well, mixed gently with diluted siRNA, and incubated for 20 min at room temperature prior to being added into a 96-well plate at 50 μL/well. Next, 50 μL of 2 × 10^5^ cells/mL was added into each well, followed by 48 h of incubation at 37°C. For transfection in a larger culture plate, the reagents were scaled up proportionally.

10.1128/mbio.01849-22.2 TABLE S2 siRNA sequences. Download Table S2, DOCX file, 0.01 MB. Copyright © 2022 Li et al. 2022 Li et al. https://creativecommons.org/licenses/by/4.0/ This content is distributed under the terms of the Creative Commons Attribution 4.0 International license.

### Generating the Caco-2-specific pro-siRNA library.

Based on our previous publications, the pro-siRNA library specifically for Caco-2 cells was produced (39, 65). Briefly, total RNAs were extracted from Caco-2 cells, followed by mRNA purification (Oligo dT beads, New England Biolabs [NEB]). The mRNAs were fragmented and converted into double-stranded DNA by reverse transcription and then second-strand synthesis (NEB). The DNA fragments were amplified by PCR and ligated into the pro-siRNA library vector pET30 (65). Library plasmids were transformed into an Escherichia coli T7 expression strain (NEB 3016). Around 1,920 E. coli colonies were cultured in 20 96-well deep-well plates for pro-siRNA production. A fraction of the E. coli culture was saved as glycerol stock for gene identification. The pro-siRNAs were purified using Ni-NTA magnetic beads in a high-throughput manner using a KingFisher Flex purification system (Thermo Fisher Scientific). After another round of DEAE purification, the final pro-siRNA library was eluted in a buffer (25 mM Tris, pH 7.0, 0.4 M NaCl, 2 mM EDTA) in 96-well plates and stored at −80°C until use.

### RNAi screen with TcdB treatment.

Caco-2 cells were transfected with siRNA (100 nM) using the Lipofectamine RNAiMAX in white opaque 96-well plates, according to the method mentioned above. Each 96-well plate included control wells with a negative-control siRNA and positive-control siRNAs targeting p22phox and UGP2. The transfected cells were incubated for 48 h, and then treated with 0.01 nM TcdB. After 18 h of intoxication, caspase activity was assayed using Caspase-Glo 3/7. We defined an apoptosis reduction greater than the standard deviation (STD) as the initial threshold for hit selection. A second screening was then performed to confirm the initial hits using both caspase assay and cell viability assay. For the hits confirmed by two rounds of screening, the pro-siRNA-producing plasmids were extracted and sequenced to reveal the identities of the candidate genes.

### Generating JUP and HMGB1 knockout cell lines.

The single guide RNA (sgRNA) sequences were cloned into LentiCRISPR V2 vector (Addgene) to target the HMGB1 and JUP genes: HMGB1 (ATTTGAAGATATGGCAAAAG) and JUP (CATGGCCTCCCGCACCCGTT). HEK293T cells were transfected with plasmids that express the sgRNA and packaging vectors of psPAX2 and pMD2.G to generate lentivirus. Caco-2 cells were infected with the lentiviruses, and puromycin (5 μg/mL) was used to select positive clones. The gene knockout cells were confirmed based on immunoblotting analysis.

### Drug treatment.

Caco-2 cells were pretreated with various concentrations of glycyrrhizin and compound glycyrrhizin injection (CGI) for 2 h before exposure to a serial concentration of TcdB for another 18 h.

### High-content analysis on cell phenotypes.

Quantitative analysis of cell phenotypes was performed using the CellInsight CX7 high-content screening (HCS) platform (Thermo Fisher Scientific). The nuclei were stained using either Hoechst 33342 (Thermo Fisher Scientific) or propidium iodine (Thermo Fisher Scientific), and the HCS Studio cell analysis software was used to determine the percentage of rounded cells (cytopathic effect) or the percentage of dead cells.

### Quantitative RT-PCR.

Total RNAs were extracted using RNAiso Plus (TaKaRa Bio). The PrimeScript cDNA synthesis kits (TaKaRa Bio) were used to convert 1 μg of total RNAs into cDNA according to the manufacturer’s instructions. Real-time PCR was performed on a Bio-Rad CFX96 real-time PCR detection system using SYBR green supermix (Bio-Rad) according to the manufacturer’s instructions. The relative expression level of each target gene was determined using gene-specific primers, and data were normalized to the expression of GAPDH (glyceraldehyde-3-phosphate dehydrogenase). The primer sequences are listed in Table S3.

10.1128/mbio.01849-22.3 TABLE S3 qPCR primer sequences. Download Table S3, DOCX file, 0.01 MB. Copyright © 2022 Li et al. 2022 Li et al. https://creativecommons.org/licenses/by/4.0/ This content is distributed under the terms of the Creative Commons Attribution 4.0 International license.

### Immunoblot assays.

To obtain whole-cell lysate, cells were washed once with phosphate-buffered saline (PBS) and then harvested with RIPA buffer (plus 100 mM phenylmethylsulfonyl fluoride [PMSF] and 1 × protease inhibitor cocktail, Thermo Fisher Scientific) and incubated for 30 min on ice. Next, the cell lysates were centrifuged at 12,000 × *g* at 4°C for 15 min to collect supernatant. For the cytochrome *c* release assay, cytosolic fractions were isolated according to a previously established method (40). To examine the release of HMGB1 from the cells into cell culture supernatants, the methanol/chloroform method was used to harvest the proteins from the culture medium (58). For the mitochondrial JUP assay, the mitochondrial fraction was obtained with a mitochondrial isolation kit (Thermo Fisher Scientific) according to the manufacturer’s instructions. The protein concentration was measured with the Pierce bicinchoninic acid (BCA) protein assay (Thermo Fisher Scientific). An equal amount of protein for each cell lysate sample was subjected to SDS-PAGE for immunoblotting.

### Mitochondrial permeabilization assays.

The mitochondrial fraction was obtained using a mitochondrial isolation kit. The mitochondrial pellet was resuspended in buffer A (250 mM sucrose, 20 mM HEPES, 10 mM KCl, 1.5 mM MgCl_2_, 1 mM EDTA, 1 mM EGTA, 1 mM dithiothreitol, 0.1 mM PMSF, pH 7.5). The 20-μL reactions were set up with 5 or 10 μL recombinant JUP (OriGene, USA) or control buffer (25 mM Tris, pH 7.3, 100 mM glycine, 10% glycerol) and 10 μL mitochondria and incubated at 37°C for 1 h. Reactions were spun at 7,000 × *g* for 10 min. The supernatant was collected and added to 6× SDS loading buffer, and the pellet was resuspended in 2× SDS loading buffer. All fractions were analyzed via Western blotting.

### Protein overexpression.

To overexpress JUP, pcDNA3-JUP or empty vector was transfected into Caco-2 cells using an SE Cell Line 4D-Nucleofector X kit (Lonza) and an Amaxa 4D Nucleofector device from Lonza. Then, 5 μg plasmid DNA was added into 5 × 10^6^ suspended cells within the kit-provided solution and electroporated using the DG-113 program in the device for Caco-2 cells according to the manufacturer’s instructions.

### Immunofluorescent staining.

Caco-2 cells were grown on coverslips in a 24-well plate. MitoTracker deep red (Thermo Fisher Scientific, M22426) was used to stain mitochondria in live cells at 37°C for 30 min. The cells were fixed with 4% paraformaldehyde for 15 min, washed with PBS, and permeabilized by 0.3% Triton X-100 for 10 min. The primary rabbit polyclonal antibody against γ-catenin (1:500) and mouse monoclonal antibody against Bcl-X_L_ (1:100) or Bax (1:100) were applied for overnight incubation at 4°C. The secondary antibodies (Alexa-Fluor 488 donkey anti-rabbit IgG and Alexa-Fluor 594 goat anti-mouse IgG) were applied at a dilution of 1:1,000 and incubated for 1 h at room temperature. Nuclei were stained with Hoechst 33342 (1:2,000). The coverslips were mounted onto glass slides with ProLong Gold antifade reagent (Thermo Fisher Scientific), and the imaging was performed on a Zeiss LSM 880 confocal microscope.

### Mouse colon loop ligation assay.

All procedures were performed according to the animal protocol approved by the Cornell University IACUC (2017-0112). C57BL/6 mice (6 to 8 weeks old; sample size indicated in Fig. 7C; female mice were used) were administered with CGI (50 mg/kg and 100 mg/kg) or control buffer daily via intraperitoneal injection 2 days before surgery. After overnight fasting, mice were anaesthetized and dissected via a midline laparotomy. An ~2-cm colon segment was ligated, and either saline or 5 μg TcdB was injected into a ligated loop. Incisions were sutured, and the mice were allowed to recover. After 8 h, mice were euthanized, and ligated colon segments were excised and subjected to H&E staining. Histological scores were blindly assessed by an independent board-certified veterinary pathologist (S.P.M.) at Cornell University.

### Statistical analysis.

*P* value was calculated using unpaired Student’s *t* test for comparison of two independent groups. One-way analysis of variance (ANOVA) was used for comparison of more than two independent groups. Statistically significant differences are marked by asterisks in the figures by *, *P* < 0.05; **, *P* < 0.01; ***, *P* < 0.001.
