# Supplementary material for: Plakoglobin and High-Mobility Group Box 1 Mediate Intestinal Epithelial Cell Apoptosis Induced by Clostridioides difficile TcdB
Source: mBio. 2022 Aug 31;13(5):e01849-22. doi: 10.1128/mbio.01849-22 (PMC9600731; doi:10.1128/mbio.01849-22)
Supplement: TABLE S1 [file mbio.01849-22-s0001.docx]

**Supplementary Tables**

**Table S1. Genes identified as involved in TcdB-mediated apoptosis**

| **No.** | **Well ID** | **Gene Name** | **Gene Description** | **Gene ID** | **Gene functions** |
| --- | --- | --- | --- | --- | --- |
| 1 | 001A6 | OGFR | Opioid growth factor receptor | 11054 | Receptor for opioid growth factor (OGF) |
| 2 | 005C5 | AHNAK | Neuroblast differentiation-associated protein AHNAK (desmoyokin) | 79026 | Nucleoprotein |
| 3 | 006D10 | HMGB1 | High mobility group box 1 | 3146 | Nuclear DNA-binding protein, transcription factor, signal molecule |
| 4 | 010D2 | JUP | Junction plakoglobin | 3728 | Cell adhesion molecule, transcription regulation |
| 5 | 014G4 | SLK | STE20 like kinase | 9748 | Mediates apoptosis and actin stress fiber dissolution |
| 6 | 014G5 | SSRP1 | Structure specific recognition protein 1 | 6749 | FACT complex subunit SSRP1, transcription regulation |
| 7 | 017H6 | ITGB1 | Integrin subunit beta 1 | 3688 | Cell adhesion molecule, cell matrix adhesion, receptor |
