# Supplementary material for: Plakoglobin and High-Mobility Group Box 1 Mediate Intestinal Epithelial Cell Apoptosis Induced by Clostridioides difficile TcdB
Source: mBio. 2022 Aug 31;13(5):e01849-22. doi: 10.1128/mbio.01849-22 (PMC9600731; doi:10.1128/mbio.01849-22)
Supplement: TABLE S2 [file mbio.01849-22-s0002.docx]

**Supplementary Tables**

**Table S2. siRNA sequences**

| **Gene Name** | **siRNA-1 sequence (5’-3’)** | **siRNA-2 sequence (5’-3’)** |
| --- | --- | --- |
| JUP | CCGUGUGUCCCAGCAAUAATT | CCAUUGUGCAUCUCAUCAATT |
| HMGB1 | GGCCCGUUAUGAAAGAGAATT | CAGGAGGAAUACUGAACAUTT |
| AHNAK | GUGCCACCAUCUACUUUGATT | GCCUAAGGCUGACGUUGUUTT |
| ITGB1 | GCAACGGACAGAUCUGCAATT | GCUCAAGCCAGAGGAUAUUTT |
| OGFR | AGGCUGGACACAGUGAGAATT | CGCCAAACCUGAGUUUCUATT |
| SLK | GCAAAUGCAGCGUUACAAUTT | GCUUCUAGAUGCCUUCUAUTT |
| SSRP1 | CCACGAGCAAAGUUGACAATT | CCAUGGACUUAAACUGCUUTT |
| UGP2 | GCAGGUACCCGAGGAUUAATT |  |
| p22phox | GCUUCACCCAGUGGUACUUTT |  |
