# Supplementary material for: Plakoglobin and High-Mobility Group Box 1 Mediate Intestinal Epithelial Cell Apoptosis Induced by Clostridioides difficile TcdB
Source: mBio. 2022 Aug 31;13(5):e01849-22. doi: 10.1128/mbio.01849-22 (PMC9600731; doi:10.1128/mbio.01849-22)
Supplement: TABLE S3 [file mbio.01849-22-s0003.docx]

**Supplementary Tables**

**Table S3. qPCR primer sequences**

| **Gene Name** | **Forward sequence (5’-3’)** | **Reverse sequence (5’-3’)** |
| --- | --- | --- |
| JUP | TGCCATAGTTGAGACGCACA | GACAAGGACGACATCACGGA |
| HMGB1 | TATGGCAAAAGCGGACAAGG | CTTCGCAACATCACCAATGGA |
| AHNAK | AGCGCATCTACACCACGAAG | CTGATGTCTATGTCCTTGGCTC |
| ITGB1 | TTGTAGCTGGTGTGGTTGCT | TGACCACAGTTGTTACGGCA |
| OGFR | GCAGGACCTACAAGGGATGA | GGTCTCCGATGGGCTCTC |
| SLK | TTAAGCGCCACGAGAAGGAAA | CTTCACTGCGCTGAATCTTGG |
| SSRP1 | ATCGTAGCTTGGGTTCATGCC | TTGTCAACTTTGCTCGTGGT |
| UGP2 | ATGTCTCAAGATGGTGCTTCTCA | GGTGTGCTCAAATTCATGTGATG |
| p22phox | CCCAGTGGTACTTTGGTGCC | GCGGTCATGTACTTCTGTCCC |
